# Intestinal Ileus as a Possible Cause of Hypobicarbonatemia

**DOI:** 10.1100/tsw.2007.39

**Published:** 2007-01-22

**Authors:** Andres Serrano, Rajani K. Chilakapati, Alexander J. Ghanayem, Yemin Yuan, Jeffery Alberts, Cathy Stephen, Giuseppe Rombola, Daniel Batlle

**Affiliations:** Division of Nephrology and Hypertension, Northwestern University Feiberg School of Medicine, Chicago, IL, USA

**Keywords:** intestinal ileus, hypobicarbonatemia, intestinal bicarbonate secretion, metabolic acidosis, hypokalemia

## Abstract

The possible occurrence of metabolic acidosis in patients with intestinal ileus is not well recognized. We describe a patient with acute alcohol-induced pancreatitis and a large transverse colon ileus in which plasma bicarbonate dropped rapidly in the absence of an increase in the plasma anion gap. The urinary anion gap and ammonium excretion were consistent with an appropriate renal response to metabolic acidosis and against the possibility of respiratory alkalosis. The cause of the falling plasma bicarbonate was ascribed to intestinal bicarbonate sequestration owing to the enhancement of chloride-bicarbonate exchange in a dilated paralyzed colon.

## INTRODUCTION

Various acid-base disturbances can occur in patients with gastrointestinal disease[1,2]. Patients with pancreatitis who present with vomiting may develop metabolic alkalosis and hypokalemia. Metabolic acidosis is usually not viewed as a complication of pancreatitis unless it is accompanied by shock or other complications. In this article, we discuss the development of metabolic acidosis in association with paralytic ileus in a patient with acute pancreatitis.

## CASE REPORT

A 45-year-old Caucasian male was admitted to the Chicago VA Hospital complaining of epigastric pain for 2 days. The pain was constant, radiated to his back, and was relieved by sitting forward. He admitted to consuming alcohol (whiskey) everyday including the day of admission. He denied vomiting, hematemesis, melena, or diarrhea. Past medical history was significant for three episodes of pancreatitis in the past. On physical examination, his temperature was 37°C, respiratory rate 18 per minute, blood pressure 138/98 mm Hg (supine) and 136/100 mm Hg (standing), and heart rate of 100 (supine) and 130 (standing) per minute. His abdomen was distended, tender to palpation, with voluntary guarding and hypoactive bowel sounds. Rectal exam revealed heme-negative stools. Workup done on admission included an abdominal radiograph that showed a large transverse colon ileus to the splenic flexure, and the following serum chemistry: sodium, 134 mEq/l; potassium, 3.0 mEq/l; chloride, 96 mEq/l; bicarbonate, 22 mEq/l; calculated anion gap, 16; blood urea nitrogen, 9 mg/dl; creatinine, 1.0 mg/dl; glucose, 123 mg/dl; calcium, 8.8 mg/dl; amylase, 313 IU (normal value 20–115 IU). Hemoglobin was 14 mg/dl and hematocrit 42.8%. Plasma ethanol level was 215 mg/dl. Urinalysis showed a specific gravity of 1.030, pH 6.0, and 4+ ketones.

A diagnosis of recurrent alcoholic pancreatitis was made; he was kept NPO and a nasogastric tube insertion was ordered, but patient refused it. During the first 12 h of hospitalization, he received 1.0 l of normal saline and 2.5 l of 5% dextrose in normal saline with 80 mEq of KCL, then he was continued on 5% dextrose in half normal saline at a rate of 150 cc/h. His urine output was 500 cc during this period. Repeated laboratory test 12 h postadmission were remarkable for an abrupt fall in serum bicarbonate with a no concurrent change in plasma anion gap (Table 1). The hematocrit has dropped from 42.9 to 37.4%. On physical exam, his abdominal distension persisted and abdominal radiographs were unchanged. Hydration was continued and 72 h after he was admitted, his general condition improved, and all the electrolytes and acid-base disturbances had normalized (Table 1). At this time, urine electrolytes were as follows: sodium, 181 mEq/l; potassium, 25 mEq/l; and chloride, 231 mEq/l. The urine anion gap was –25 mEq/l. Urine pH, 6.1; ammonium, 41 mEq/l; and titratable acidity, 11 mEq/l. Fractional bicarbonate excretion was 0.4%.

**TABLE 1**

| Serum Level | At Admission | 12 h | 24 h | 72 h |
|---|---|---|---|---|
| Sodium (mEq/l) | 134 | 134 | 137 | 131 |
| Potassium (mEq/l) | 3.0 | 3.8 | 4.0 | 3.5 |
| Chloride (mEq/l) | 96 | 101 | 104 | 94 |
| Total $CO_2$ (mEq/l) | 22 | 14 | 17 | 25 |
| Anion gap (mEq/l) | 16 | 19 | 16 | 12 |
| Urea nitrogen (mg/dl) | 9.0 | 12 | 9.0 | 4.0 |
| Creatinine (mg/dl) | 1.0 | 1.1 | 1.2 | 0.9 |
| Hematocrit (mg/dl) | 42.9 | 37.4 | 37.5 | 42.8 |

## DISCUSSION

The acid-base disturbance that developed in our patient 12 h following admission could be a primary metabolic acidosis, a primary respiratory alkalosis with metabolic compensation, or a combination of both (i.e., a mixed metabolic acidosis and respiratory alkalosis). An arterial blood gas analysis is clearly needed to establish the cause of hypobicarbonatemia in our patient. However, our patient declined to have one due to previous painful experiences in the past. Even in the absence of this information, we believe that it is highly improbable that our patient had a primary respiratory alkalosis.

A primary respiratory alkalosis would be expected to elicit a compensatory decrease in bicarbonate concentration of about 0.5 mEq/l per each 1 mm Hg decrement in $pCO_2$ after about 48 h of hyperventilation[3]. In our patient, in whom plasma bicarbonate dropped by 8 mEq/l (from 22 to 14 mEq/l) over a period of only 12 h (Table 1), blood $pCO_2$ would have to have fallen by about 16 mm Hg to produce the observed fall in plasma bicarbonate. This possibility is unlikely insofar as there was no change in the patient’s clinical condition (absence of fever, sepsis, liver failure, etc.) and there was insufficient time for the kidney’s full adaptive response to respiratory alkalosis. Furthermore, after recovery from chronic hypocapnia, urinary acidification remains suppressed for sometime and this is manifested by a decrease in acid excretion[4]. However, our patient had abundant ammonium in his urine, reflected in a negative urinary anion gap, which essentially excludes a primary respiratory alkalosis[1,6]. Based on all these considerations, a primary metabolic acidosis with an appropriate increase in renal acid excretion appears the most likely cause of hypobicarbonatemia in our patient. The only source of $HCO_3$ loss that could be suspected in this patient was the sequestration of $HCO_3$ in the dilated intestinal loops owing to paralytic ileus after pancreatitis.

In a pure high anion gap metabolic acidosis, the fall in plasma bicarbonate is matched by an identical rise in the anion gap[5]. The elevation in the plasma anion gap, which was present on arrival to the hospital, was ascribed to the formation of ketoanions as a result of alcohol abuse and starvation. At 12 and 24 h following admission, however, the anion gap was elevated, but not sufficiently as to account for the observed decrement in total $CO_2$ content (Table 1). Alcoholic ketoacidosis is usually of the high anion gap variety[5]. In fact, contrary to the findings in our patient, many of the patients with ketoacidosis usually present with an increment in the anion gap that exceeds the decrement in plasma bicarbonate probably because of generation of bicarbonate from vomiting. The abrupt fall in plasma bicarbonate that occurred while the patient received fluids and in the absence of a concurrent increase in the anion gap was, therefore, ascribed to the development of a hyperchloremic type of metabolic acidosis superimposed on the initial ketoacidosis.

We would like to propose that the falling bicarbonate level was caused by the presence of intestinal ileus. The common causes of hyperchloremic metabolic acidosis were reasonably excluded by history. The patient did not receive any drugs or acid loads containing $Cl^-$ as the anion, and did not have an ureteroenterostomy. The development of a biliary, pancreatic, or intestinal fistula is unlikely considering that the patient promptly recovered to his normal state of health. The urine anion gap was negative, suggesting that the patient's urine contained adequate amounts of ammonium[6]. This was verified by direct measurement of ammonium in urine, which was increased, making the diagnosis of distal renal tubular acidosis unlikely. Renal bicarbonate wastage can be excluded because the fractional excretion of bicarbonate was less than 1% when plasma bicarbonate had normalized. Dilution of the extracellular volume compartment is usually cited as a cause of hyperchloremic metabolic acidosis[5]. Although frequently invoked, however, dilutional acidosis is rarely, if ever, a cause of metabolic acidosis in the clinical setting. As reviewed by Garella et al.[7], an extracellular fluid expansion of as much as 38% would be necessary to result in a decrease in bicarbonate concentration of 2 mEq/l. Accordingly, volume expansion could not account for the marked fall in bicarbonate concentration in our patient in whom the extracellular volume was expanded by only 12.6%. *Note:* This calculation is based on the fall in plasma hematocrit noted 12 h following arrival to the hospital:

$$\frac{(42.9 - 37.6) \times 100}{42.9} = 12.6\%$$

The presence of a severe intestinal ileus with sequestration of fluid within the bowel prompted us to consider the possibility of internal gastrointestinal bicarbonate losses as the cause of hyperchloremic metabolic acidosis. Since our patient did not have any bowel movements for at least 72 h, diarrhea is obviously excluded as a cause of bicarbonate loss. In the colon, like in the ileus, there is $Na^+$ and $Cl^-$ absorption, and active secretion of $HCO_3$[1]. A $Cl^-/HCO_3$ exchanger mediates chloride absorption and bicarbonate secretion[13,14] (Fig. 1). In the face of an intestinal ileus, the accumulation of fluid in the bowel, coupled with an increase in functional surface area, augments chloride/bicarbonate exchange, a process that could lead to bicarbonate loss into the intestinal lumen and, thus, metabolic acidosis.

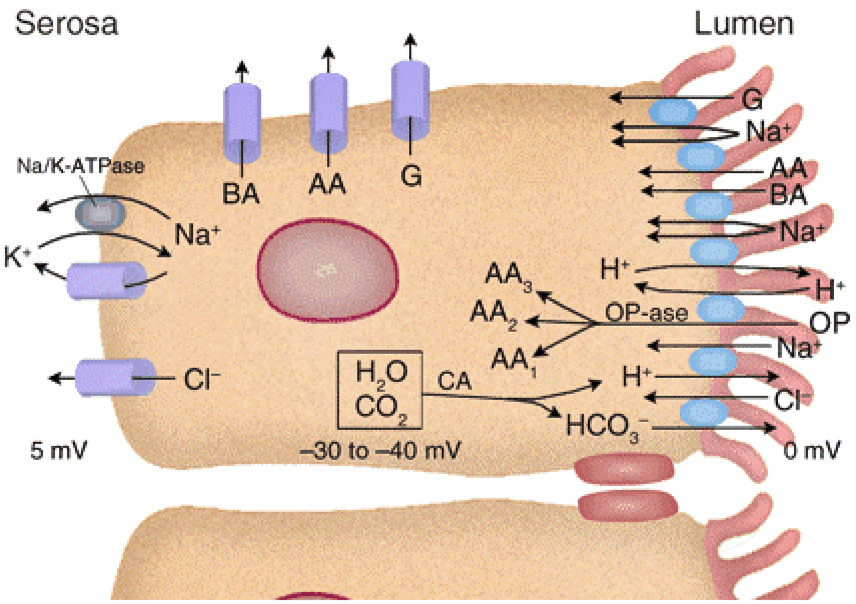

**FIGURE 1**. Ileal absorptive cell, multiple-brush border transporters couple ion influxes ($Na^+$ and $H^+$) to organic solute influxes or exchange for bicarbonate; cellular $H^+$ and $HCO_3^-$ extruded in exchange for $Na^+$ and $Cl^-$ is provided through the action of carbonic anhydrase (CA). Additional $HCO_3^-$ may enter the cell through the basolateral membrane NHE1 or the $Na/HCO_3$ cotransporter (not shown). (Reprinted from Field[14] with permission.)

An increase in net chloride reabsorption, however, is also predicted from the mode of operation of the $Cl^-/HCO_3$ exchanger. Experimental work has shown that when intraluminal pressure is increased, the driving forces for filtration secretion are augmented[8,9,10]. Further, active solute transport mechanisms may be impaired in patients with ileus, as it has been shown experimentally when intraluminal pressures are increased[7,8]. These processes, coupled with the increase in effective surface area and possibly an increase in vascular permeability often seen in patients with pancreatitis[11], leads to solute and fluid accumulation within the intestinal lumen. Accordingly, the transfer of sodium chloride into the intestinal lumen increases even though the active operation of $Cl^-/HCO_3$ exchanger results in chloride resorption. Thus, until the ileus resolves, there is progressive accumulation of solute and water in the intestinal lumen despite active $Cl^-/HCO_3$ exchange. This leads to two different effects: volume depletion and metabolic acidosis. We believe that, in our patient, the former was corrected faster than the latter with the administration of sodium chloride and water. Correction of the metabolic acidosis required enhancement of renal acid excretion as seen in our patient. Although not commonly recognized and, in fact, not mentioned in standard nephrology texts[12], intestinal ileus should be considered in the differential of hyperchloremic metabolic acidosis. We conclude that internal bicarbonate losses into dilated colonic loops need to be considered as a cause of metabolic acidosis in patients with abdominal ileus.
